# Amentoflavone Affects Epileptogenesis and Exerts Neuroprotective Effects by Inhibiting NLRP3 Inflammasome

**DOI:** 10.3389/fphar.2019.00856

**Published:** 2019-07-30

**Authors:** Shikuo Rong, Ding Wan, Yayun Fan, Shenhai Liu, Kuisheng Sun, Junming Huo, Peng Zhang, Xinxiao Li, Xiaoliang Xie, Feng Wang, Tao Sun

**Affiliations:** ^1^Ningxia Key Laboratory of Cerebrocranial Disease, Incubation Base of National Key Laboratory, Ningxia Medical University, Yinchuan, China; ^2^Department of Neurosurgery, General Hospital of Ningxia Medical University, Yinchuan, China; ^3^Department of Gynaecology, Jingzhou Central Hospital affiliated to Huazhong University of Science and Technology, Jingzhou, China

**Keywords:** epileptogenesis, inflammation, amentoflavone, NLRP3 inflammasome, neuroprotection

## Abstract

Brain inflammation is one of the main causes of epileptogenesis, a chronic process triggered by various insults, including genetic or acquired factors that enhance susceptibility to seizures. Amentoflavone, a naturally occurring biflavonoid compound that has anti-inflammatory effects, exerts neuroprotective effects against nervous system diseases. In the present study, we aimed to investigate the effects of amentoflavone on epilepsy *in vivo* and *in vitro* and elucidate the underlying mechanism. The chronic epilepsy model and BV2 microglial cellular inflammation model were established by pentylenetetrazole (PTZ) kindling or lipopolysaccharide (LPS) stimulation. Cognitive dysfunction was tested by Morris water maze while hippocampal neuronal apoptosis was evaluated by immunofluorescence staining. The levels of nucleotide oligomerization domain-like receptor protein 3 (NLRP3) inflammasome complexes and inflammatory cytokines were determined using quantitative real-time polymerase chain reaction, Western blotting, immunofluorescence staining, and enzyme-linked immunosorbent assay. Amentoflavone reduced seizure susceptibility, minimized PTZ-induced cognitive dysfunction, and blocked the apoptosis of hippocampal neurons in PTZ-induced kindling mice. Amentoflavone also inhibited the activation of the NLRP3 inflammasome and decreased the levels of inflammatory cytokines in the hippocampus of PTZ-induced kindling mice. Additionally, amentoflavone could alleviate the LPS-induced inflammatory response by inhibiting the NLRP3 inflammasome in LPS-induced BV2 microglial cells. Our results indicated that amentoflavone affects epileptogenesis and exerts neuroprotective effects by inhibiting the NLRP3 inflammasome and, thus, mediating the inflammatory process in PTZ-induced kindling mice and LPS-induced BV2 microglial cells. Therefore, amentoflavone may be a potential treatment option for epilepsy.

## Introduction

Epilepsy, a common chronic brain disorder and the most common severe neurological condition (Loscher et al., 2013; Lukawski et al., 2016), affects ∼1% of the population and all ages. Epilepsy often requires lifelong medication (Hauser et al., 2018), and as more than 30% of patients suffering from this disorder do not achieve good control with available drugs, this condition often progresses to drug-resistant epilepsy (Laxer et al., 2014). Therefore, developing new drugs to prevent or treat epilepsy is still urgently needed. Epileptogenesis is a chronic process triggered by various insults including genetic or acquired factors, thereby enhancing one’s susceptibility to seizures, and ultimately generating spontaneous recurrent seizures (Goldberg and Coulter, 2013; Pitkanen et al., 2015). Inflammatory processes are associated with the etiology and clinical progression of epileptogenesis (Singhi, 2011; Ngugi et al., 2013) and lead to aberrant neural connectivity and hyper-excitable neuronal network, which mediate epileptogenesis (Rana and Musto, 2018). Anti-inflammatory therapies may serve as an intervention and could be a promising strategy for preventing and treating seizures and its related neurobehavioral comorbidities.

Inflammasome, a multi-protein complex that typically consists of an adaptor protein apoptosis-associated speck-like protein (ASC) and pro-caspase-1, is the key platform of inflammatory signaling pathway that detects pathogenic microorganisms and sterile stressors and activates the pro-inflammatory cytokines, interleukin-1β (IL-1β), and interleukin-18 (IL-18) (Schroder and Tschopp, 2010). Inflammasome-mediated processing and secretion of pro-IL-1β and pro-IL-18 enable a rapid, yet tightly regulated and highly inductive pro-inflammatory response. The activation of NLRP3 (Heneka et al., 2013; Heneka et al., 2014; Hughes and O’Neill, 2018), nucleotide oligomerization domain (NOD)-like receptor protein 1 (NLRP1) (Bernales et al., 2018), and NLR family CARD domain containing 4 (NLRC4) (Duncan and Canna, 2018), and the absence of Melanoma 2 (AIM2) (Denes et al., 2015) inflammasomes are all associated with various neurological diseases. As the most classic inflammasome, NLRP3 is involved in many neurological diseases such as Alzheimer’s disease (Heneka et al., 2013), cerebrovascular disease (Shao et al., 2015; Lenart et al., 2016), and epilepsy (Meng et al., 2014). Not only is the NLRP3 inflammasome the best studied inflammasome, it is also implicated in inflammation and epilepsy (Meng et al., 2014; Jang et al., 2016; Lenart et al., 2016; Wang et al., 2017). When NLRP3 is inhibited, this causes antiepileptic and neuroprotective effects following amygdala kindling-induced status epilepticus (Meng et al., 2014). NLRP3 inflammasome deficiency also protects amyloid precursor protein/presenilin-1 (APP/PS1) mice from loss of cognitive function (Heneka et al., 2013). These findings strongly suggest that NLRP3 inflammasome inhibition represents a new therapeutic target for epilepsy.

Amentoflavone is a natural biflavone compound with many biological properties, including anti-inflammatory, anti-oxidative, and anti-apoptotic effects (Oh et al., 2013; Cao et al., 2017). Our previous experimental results found that amentoflavone also exerts neuroprotective effect and prevents the occurrence of seizures by inhibiting inflammatory processes (Zhang et al., 2015). However, the underlying mechanisms and functions of amentoflavone on epilepsy have not been fully explored. In the present study, antiepileptic and neuroprotective effects of amentoflavone were investigated in PTZ-induced kindling, a good simulation of epileptogenesis, in mice with epilepsy. To determine amentoflavone’s related mechanism of action, we examined amentoflavone’s effect on NLRP3 inflammasome activation and the associated inflammatory processes in PTZ-induced kindling mice and LPS-induced BV2 microglial cells.

## Materials and Methods

### Animals

Male C57BL/6 mice (6–8 weeks old) were obtained from the Experimental Animal Center of Ningxia Medical University. Animals were housed in a specific pathogen-free (SPF) environment under a 12-h light/dark cycle with free access to food and water. The study was approved by the Ningxia Key Laboratory of Cerebrocranial Disease, Incubation Base of National Key Laboratory of Ningxia Medical University, and the protocol for the mice study was approved by the Animal Research Ethics Committee of Ningxia Medical University (2014-143).

### Cell Lines and Culture Conditions

Mouse microglial cell line, BV2, was purchased from the National Infrastructure of Cell Line Resource (Beijing, China) and cultured in Dulbecco’s modified Eagle’s medium (DMEM; Gibco, MD, USA) containing 10% fetal bovine serum (FBS; Gibco, MD, USA) at 37°C in 5% CO_2_.

BV2 microglial cells were divided into four groups: LPS group, administered LPS [1 µg/ml (Nam et al., 2018)]; LPS+AF group, administered amentoflavone (10 μM) followed by LPS (1 µg/ml); AF group, administered amentoflavone alone (10 μM); and Control group, administered dimethyl sulfoxide (DMSO, 1%) followed by PBS.

### Chemicals and Antibodies

Amentoflavone and PTZ were purchased from Sigma-Aldrich (St. Louis, MO, USA). Amentoflavone purity was ≥99.0%; its chemical formula is C_30_H_18_O_10_. Primary antibodies of NLRP3 (bs-10021R), ASC (bs-6741R), caspase-1 p20 (bs-10442R), IL-18 (bs-4988R), IL-1β (bs-0812R), and β-actin (bs-0061R) were purchased from Bioss Biotechnology Co., Ltd. (Woburn, MA). Primary antibodies of Bcl-2 (ab59348), Bax (ab32503), cleaved caspase-3 (ab2302), TNF-α (ab6671), and Goat Anti-Rabbit IgG H&L (Alexa Fluor^®^ 488) (ab150077) were purchased from Abcam (San Francisco, CA, USA). Rabbit IgG (H&L) Secondary antibody IRDye800CW Conjugated was purchased from LI-COR Bioscience (Lincoln, NE, USA). Other general agents were commercially available.

### Model for the Kindling Procedure

Mice were intraperitoneally (i.p.) injected with PTZ (37 mg/­kg) once every other day for a total of 15 injections. Three consecutive stage 4 seizures indicated full kindling. Behavior of mice was observed for 30 min after each injection and seizure intensity was scored as follows: stage 0, no response; stage 1, facial movements, ear, and whisker twitching; stage 2, convulsive waves throughout the body; stage 3, myoclonic convulsions with rearing; stage 4, clonic–tonic convulsions; stage 5, generalized clonic–tonic seizures with loss of postural control; or death.

Mice were randomly divided into five groups: PTZ group, administered PTZ (37 mg/kg, i.p.) 2 h after intragastric vehicle (1% DMSO, p.o.) administration once every other day for a total of 15 injections (29 days); PTZ+AF group, administered amentoflavone [25 mg/kg p.o. concentration based on our previous research results (Zhang et al., 2015)] at 2 h prior to PTZ administration once every other day; AF group, administered amentoflavone alone (25 mg/kg, p.o.) once every other day; CON-1 group (for comparison to PTZ group), administered saline (0.9% NaCl, i.p.); and CON-2 group (for comparison to PTZ+AF group), administered vehicle (1% DMSO, p.o.). Amentoflavone and PTZ were prepared separately before each administration.

### Electroencephalogram Measurement

The electroencephalic electrode was implanted 10 days before the injection of PTZ or vehicle. After anesthetizing mice with 1% pentobarbital, electrodes were implanted into specific locations in the cortex using a stereotactic instrument. Coordinates were set for the recording electrode (coordinates from the bregma: 1.5 mm lateral and 2.0 mm posterior to the bregma and left 1.2 mm below the dura) and reference electrode (1.5 mm lateral and 2.0 mm posterior to the bregma and right 1.2 mm below the dura). After the last PTZ administration, electrocorticography (ECoG) was recorded by an acquiring and processing system of biomedical signals (BL-420 N, Techman Software, Chengdu, China).

### Morris Water Maze Test

Twenty-four hours following the final PTZ administration, the Morris Water Maze (MWM) test was performed to determine the behavior and cognitive ability of mice. MWM is composed of a black circular pool (diameter 120 cm, height 50 cm) filled with water (depth 20 cm, temperature 21°C) and a circular platform (diameter 10 cm) for animals to escape. The pool was divided into four equal quadrants, and the escape platform was placed in a constant quadrant (target quadrant) and submerged 1 cm below the water surface. White pigment was added to the water followed by mixing to achieve an opaque appearance. The reference around the pool remained the same. For acclimation to the pool environment, mice were allowed 2 min to freely swim in the pool on the day before the experiment.

The MWM test consisted of three parts: Place navigation test, Spatial probe test, and Visible platform trial. Place navigation test was first used to assess the spatial learning ability of mice for 5 days. Mice were trained four times per day and divided into four water inlet points with their head facing the wall of the pool according to quadrants I, II, III, and IV, respectively. Escape latency (the ability to climb onto a platform within 60 s) was then recorded with a 15-min interval in each training. Mice were placed on the platform for 15 s; if unable to find the underwater platform within 60 s, escape latency was recorded as 60 s. Spatial probe test was used to determine the spatial memory ability of mice. The hidden platform was removed on the second day after place navigation test. Mouse was released from the quadrant opposite to the target quadrant and then allowed to swim freely for 60 s; the time of swimming in the quadrant with the former platform and crossing from the former platform area were recorded. After the probe trial, visual platform experiment was used to eliminate the influence of animal vision and motor function on spatial learning and memory ability of mice. The escape platform was raised 1.5 cm above water level. Escape latency and swimming speed were recorded. After each experiment, mice were allowed to dry and acclimate to a normal temperature, before being returned to their home cage.

### Immunofluorescence Staining

Preparation of tissue sections: Twenty-four hours after the last injection of PTZ, mice were anesthetized and intracardially perfused with 0.9% saline and 4% paraformaldehyde. Their brains were then quickly removed and fixed in 4% paraformaldehyde. Specimens were dehydrated with sucrose, frozen, and then soaked in PBS solution for 10 min.

Preparation of BV2 microglial cells: BV2 microglial cells were treated with amentoflavone or vehicle (1% DMSO) for 30 min, and then treated with LPS (1 µg/ml) or PBS for 5.5 h. Cells were then fixed with 4% paraformaldehyde for 10 min and washed three times with PBS.

Tissue sections and BV2 microglial cells were treated with 3% hydrogen peroxide and incubated with 3% bovine serum albumin (BSA) followed by anti-cleaved caspase-3 (1:1,000) or anti-NLRP3 (1:500), ASC (1:500), and caspase-1 p20 (1:500) overnight at 4°C. Sections were subsequently incubated with Goat Anti-Rabbit IgG H&L (Alexa Fluor^®^ 488) (1:500) for 1 h at room temperature. Cells were mounted with a sealer containing DAPI (ZLI-9557, ZSGB-BIO, Beijing, China), and images were captured with a Leica DM6 fluorescence microscope (Leica, Germany).

### Quantitative Real-Time Polymerase Chain Reaction

Twenty-four hours after the last injection of PTZ, total RNA was extracted from hippocampal samples using TRIzol^™^ reagent (15596026, Invitrogen). Five hundred nanograms of total RNA was reverse-transcribed using Revert Aid First Strand cDNA Synthesis Kit (Thermo Scientific, K1621), and the synthesized cDNA was used for real-time polymerase chain reaction (PCR) with a Bestar^®^ Sybr Green qPCR Master Mix (DBI-2043, DBI Bioscience, Shanghai, China). PCR amplifications of cDNA were performed on a CFX-96 PCR system (Bio-Rad, Hercules, CA, USA) using standard methods. Primer sequences for NLRP3, ASC, and caspase-1 were based on a published report (Huai et al., 2014), and primer synthesis was performed by Sangon Biotech (Shanghai). Ct values were calculated and analyzed by the 2^−ΔΔCt^ method to evaluate the relative transcript levels on PRISM 6 (GraphPad software, La Jolla, CA, USA). All experiments were performed in triplicate.

### Western Blot Analysis

Total protein from the hippocampal tissue and BV2 microglial cells was prepared and extracted using the BCA Protein Extraction Kit (KGP2100, KeyGEN Biotechnology Co., Ltd, Jiangsu, China). Protein concentration was measured using the BCA Protein Assay Kit (KGP902, KeyGEN Biotechnology Co., Ltd, Jiangsu, China). Equal amounts of protein (50 µg per lane) were resolved on a 10% or 12% sodium dodecyl sulfate–polyacrylamide gel (SDS-PAGE), and then transferred onto 0.22-μm polyvinylidene fluoride (PVDF) membranes (Millipore, USA) blocked with 5% non-fat milk, and incubated with the following primary antibodies overnight at 4°C: rabbit anti-Bcl-2 (1:500), Bax (1:1000), cleaved caspase-3 (1:1,000), NLRP3 antibody (1:500), caspase-1 p20 (1:500), ASC (1:500), IL-18 (1:500), IL-1β (1:500), TNF-α (1:1,000), and β-actin (1:2,000). Membranes were washed with TBST (TBS containing 1‰ Tween 20) and subsequently incubated with Goat Anti-Rabbit IgG (H&L) Secondary antibody Conjugated (1:5,000). Detection was performed with an Odyssey Infrared Imaging System CLX-0796 (LI-COR, Lincoln, NE, USA) and quantified *via* densitometry with Image-Pro Plus 6.0 software (Media Cybernetics, Inc., Rockville, MD, USA). All experiments were performed in triplicate.

### Enzyme-Linked Immunosorbent Assay

Twenty-four hours after the last injection of PTZ, protein samples of the hippocampus of mice were rinsed, homogenized, and stored overnight at −20°C. After two freeze–thaw cycles, the homogenates were centrifuged. The protein concentration was determined as described above. In addition, the supernatant of each group of BV2 microglial cells was also collected. Then, IL-1β, IL-18, and TNF-α concentrations were measured with a specific enzyme-linked immunosorbent assay (ELISA) kit (Cusabio Biotech, Wuhan, China) according to the manufacturer’s protocol. Absorbance was measured at 540 nm with a microplate reader.

### MTT Assay

BV2 microglial cells were seeded into 96-well plates at a density of 4 × 10^3^ per well with amentoflavone at different concentrations (50 nM to 1 µM for lower doses and 5 to 150 µM for higher doses) in the absence of FBS. After 24 h, cell viability was determined by an MTT assay. In brief, 5 mg/ml of MTT was added for a 4-h cell incubation at 37°C. Cell supernatant was then removed and 200 µl of DMSO was added to dissolve the crystal. Optical density was detected with a microplate reader at 580 nm.

### Statistical Analysis

Statistical analysis was performed using PRISM 6 software. Results are presented as mean ± standard deviation (SD). Two-way analysis of variance (ANOVA) with repeated measures followed by Bonferroni or Dunnett’s T3 *post hoc* test was used to analyze data related to seizure stage and escape latency in MWM. Student’s *t* test and one-way ANOVA were used for comparisons between and among different groups. At least three independent experiments were performed for each condition.

## Results

### Amentoflavone Reduces Seizure Susceptibility in PTZ-Induced Kindling Mice

**Figure 1:**
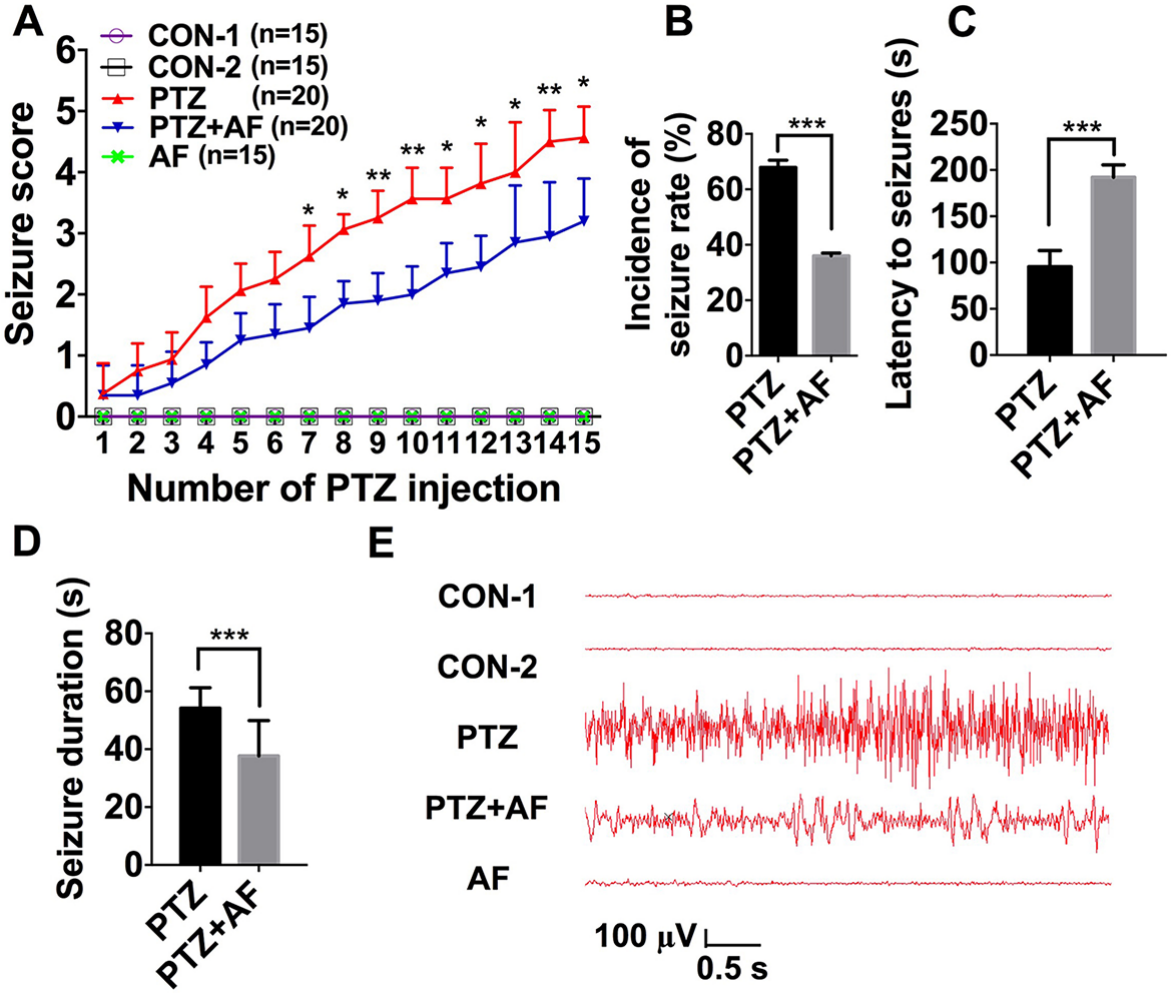
Effects of amentoflavone on seizure susceptibility in PTZ-induced kindling in mice. **(A)** Statistical results showing the decrease in seizure score due to amentoflavone treatment (two-way ANOVA with repeated measures followed by *Bonferroni* or *Dunnett’s T3 post hoc test*). **(B)** Statistical results showing the decrease in the incidence of full kindling due to amentoflavone treatment (*chi-square test*). **(C)** Statistical results showing the increase in latency to generalized seizures due to amentoflavone treatment (*Student’s t test*). **(D)** Statistical results showing the decrease in duration of generalized seizures due to amentoflavone treatment (*Student’s t test*). **(E)** Representative EEG recording of approximately 2 h of PTZ-induced seizure. Error bars represent mean ± s.d. *, **, *** represent*p* < 0.05, *p* < 0.01, *p* < 0.001, respectively. PTZ, pentylenetetrazole; AF, amentoflavone.

First, we investigated the effect of amentoflavone on the susceptibility to epileptic seizures. Seizure stage score (stage 4 or greater), incidence of seizure, latent period (duration from PTZ injection to seizure event), and seizure duration were used to evaluate seizure susceptibility. Compared to the PTZ group, amentoflavone (25 mg/kg) treatment decreased the seizure stage score from 4.56 ± 0.50 to 3.2 ± 0.68 (*p* < 0.001; **Figure 1A**) and incidence of full kindling from 68.75% to 35.00% in PTZ-induced kindling mice (**Figure 1B**). However, amentoflavone alone did not affect the susceptibility to epileptic seizures in mice (*p* > 0.05; **Figure 1A**), but it significantly increased the latency of seizures and reduced the duration of seizures (*p* < 0.001; **Figures 1C, D**). To verify whether the behavior of seizures was consistent with electroencephalogram (EEG), cortical electrodes were implanted into mice to record seizures. From the results, the behavioral performance of seizures induced by PTZ kindling synchronized with EEG performance. As expected, compared to the PTZ mice, mice treated with amentoflavone had lower frequency and amplitude of electrographic seizure activities (**Figure 1E**). These results suggest that amentoflavone reduces seizure susceptibility and exerts antiepileptic seizure activity in PTZ kindled mice.

### Amentoflavone Minimizes PTZ-Induced Cognitive Dysfunction in Mice

**Figure 2:**
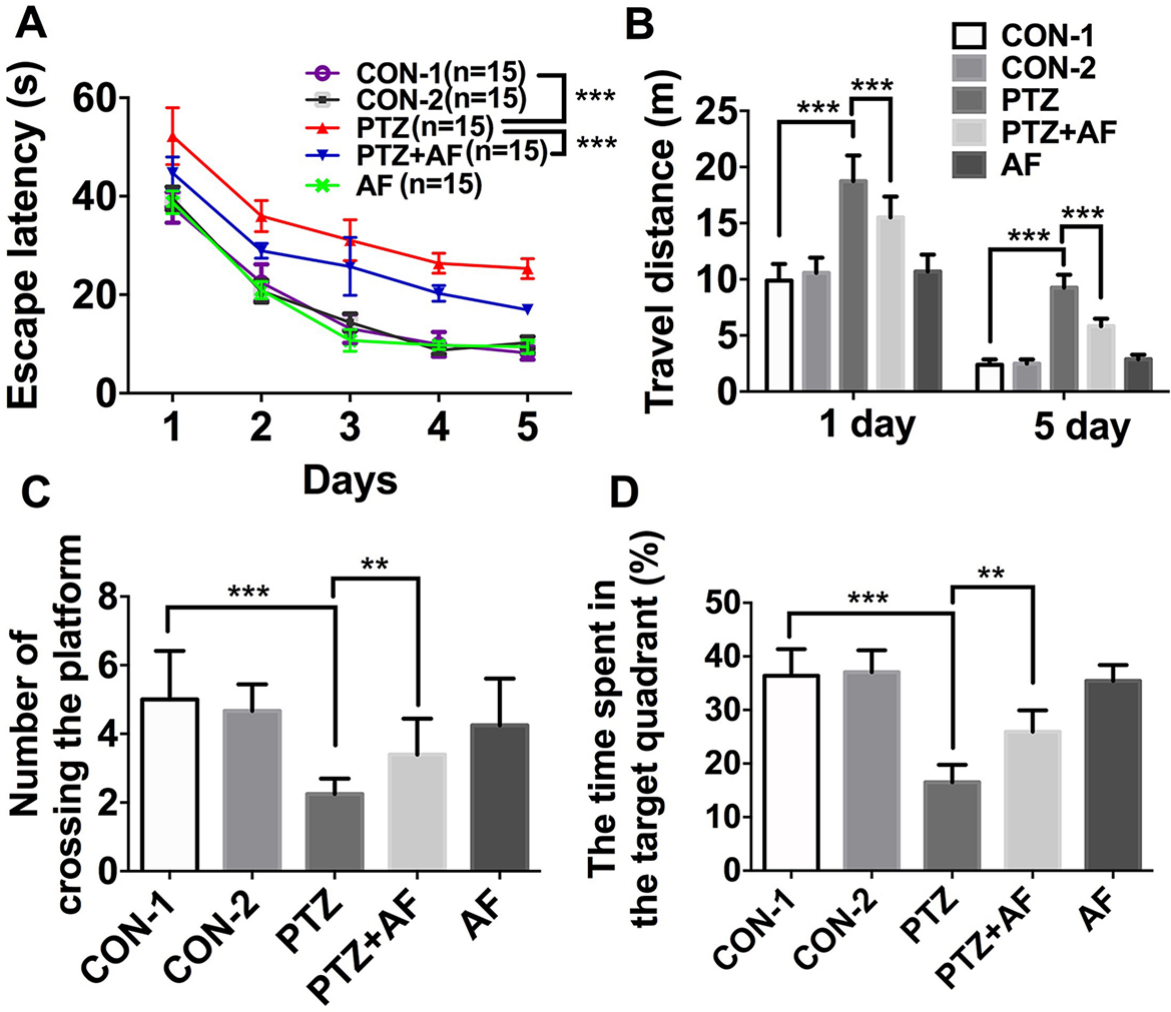
Effects of amentoflavone on cognitive deficits induced by PTZ kindling. **(A)** Statistical results showing the decrease in escape latency by amentoflavone (two-way ANOVA with repeated measures followed by *Bonferroni* or *Dunnett’s T3 post hoc test*). **(B)** Statistical results showing the decrease in swimming distance by rats in search of the hidden platforms due to amentoflavone treatment (*Student’s t test*). **(C)** Statistical results showing the increase in the number of rats crossing the target quadrant due to amentoflavone treatment (*Student’s t test*). **(D)** Statistical results showing the increase in the time spent in the target quadrant due to amentoflavone treatment (*Chi-square test*). Error bars represent mean ± s.d. **, *** represent *p* < 0. 01, *p* < 0.001, respectively. PTZ, pentylenetetrazole; AF, amentoflavone.

MWM was used to test cognitive dysfunction in mice with PTZ-induced kindling. In the place navigation test, escape latency for each group gradually decreased over the five training days and was significantly longer in the PTZ group than the control group. However, compared to the PTZ group, amentoflavone (25 mg/kg) treatment significantly reduced escape latency (*p* < 0.001; **Figure 2A**). On the fifth training day, mice in the PTZ group had to travel a longer distance to reach the hidden platform than those in the control group. However, by amentoflavone (25 mg/kg) treatment, there was a significant decrease in their swimming distance to find the hidden platform (*p* < 0.001; **Figure 2B**). In the Spatial probe test, the number of mice crossing the target quadrant and the time spent in the target quadrant within 60 s were significantly lower in the PTZ group than in the control group. Indeed, amentoflavone treatment significantly increased the number of mice crossing the target quadrant and the time spent in the target quadrant compared to those of the PTZ group (*p* < 0.01; **Figures 2C, D**). Amentoflavone alone did not influence escape latency, travel distance, number of mice crossing the target quadrant, and time spent in the target quadrant. In fact, there was no difference between the group treated with amentoflavone alone and the control group (*p* > 0.05; **Figure 2**). These data confirm that amentoflavone minimizes PTZ-induced cognitive dysfunction in mice.

### Amentoflavone Blocks Apoptosis of Hippocampal Neurons in PTZ-Induced Kindling Mice

**Figure 3:**
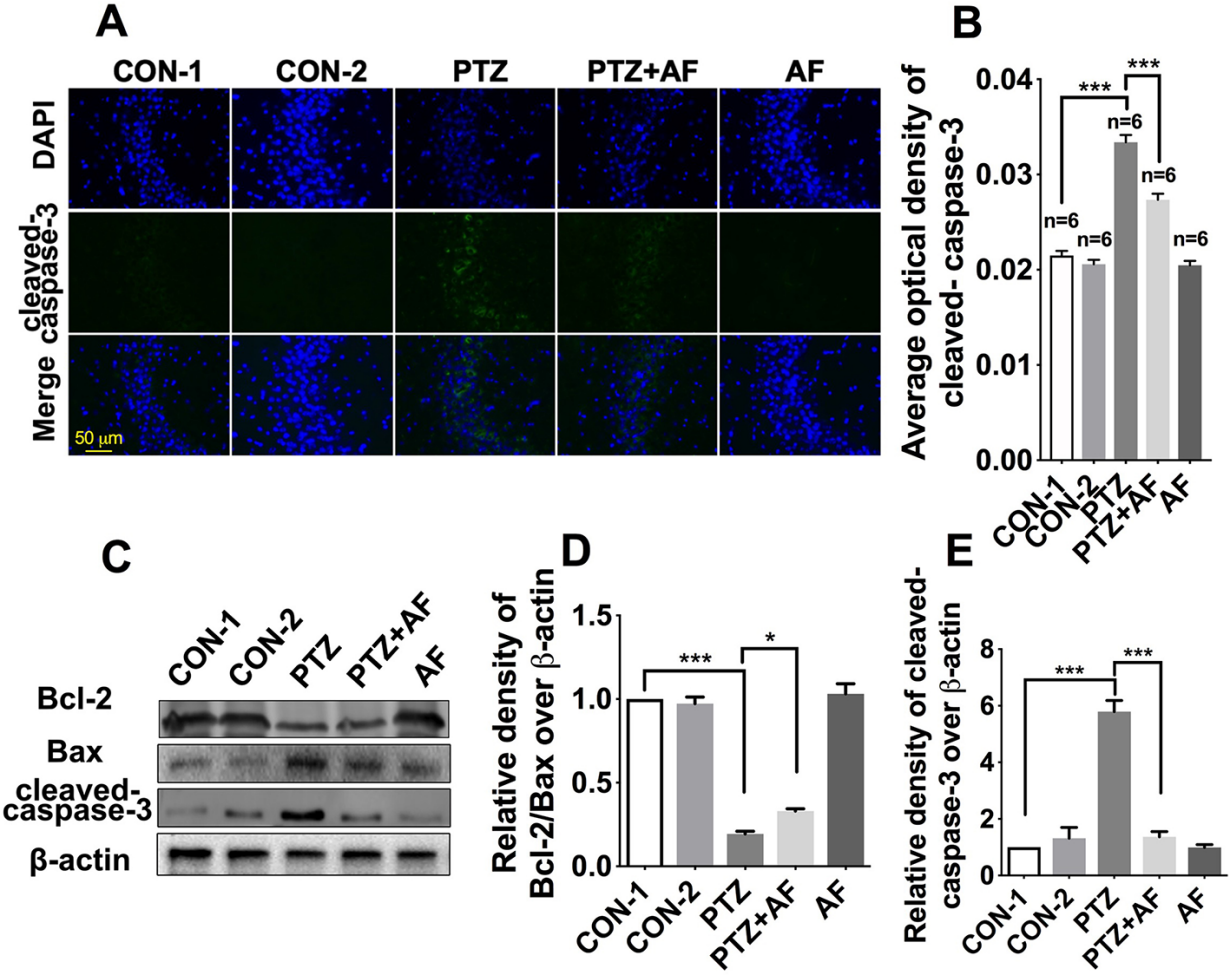
Effects of amentoflavone on hippocampal neuronal apoptosis induced by PTZ kindling. **(A)** Representative images of immunofluorescence staining of cleaved caspase-3 in the hippocampus. **(B)** Semi-quantitative analysis of the relative levels of cleaved caspase-3 by densitometric analysis (*n* = 6 mice per group, *Student’s t test*). **(C)** Representative immunoblots of Bcl-2, Bax, and cleaved caspase-3 proteins in different groups. **(D** and **E)** Semi-quantitative analysis of the relative levels of Bcl-2/Bax and cleaved caspase-3 by densitometric analysis (*n* = 6 mice per group, *Student’s t test*). Error bars represent mean ± s.d.*, *** represent *p* < 0.05, *p* < 0.001, respectively. PTZ, pentylenetetrazole; AF, amentoflavone.

Apoptosis is well known as an important feature of epilepsy. First, we examined apoptosis in hippocampal neurons using cleaved caspase-3 *via* immunofluorescence staining. The results showed that cleaved caspase-3 appeared in the cytoplasm, and its fluorescence intensity was significantly enhanced by PTZ-induced kindling. Indeed, treatment with amentoflavone significantly reduced the fluorescence intensity caused by PTZ-induced kindling (*p* < 0.001; **Figure 3B**). There was no obvious fluorescence in the amentoflavone alone group and the control group (**Figure 3A**). Neuronal apoptosis was evaluated by Western blot analysis (**Figure 3C**). This revealed that amentoflavone treatment alone could significantly increase the ratio of Bcl-2/Bax and decrease the expression of the apoptosis-related protein, cleaved caspase-3, that were changed by PTZ-induced kindling in the hippocampus (*p* < 0.05; **Figures 3D, E**); these results are consistent with those derived from immunofluorescence staining. The above data show that amentoflavone blocks hippocampal neuronal apoptosis due to PTZ kindling.

### Amentoflavone Inhibits NLRP3 Inflammasome Activation in PTZ-Induced Kindling Mice

**Figure 4:**
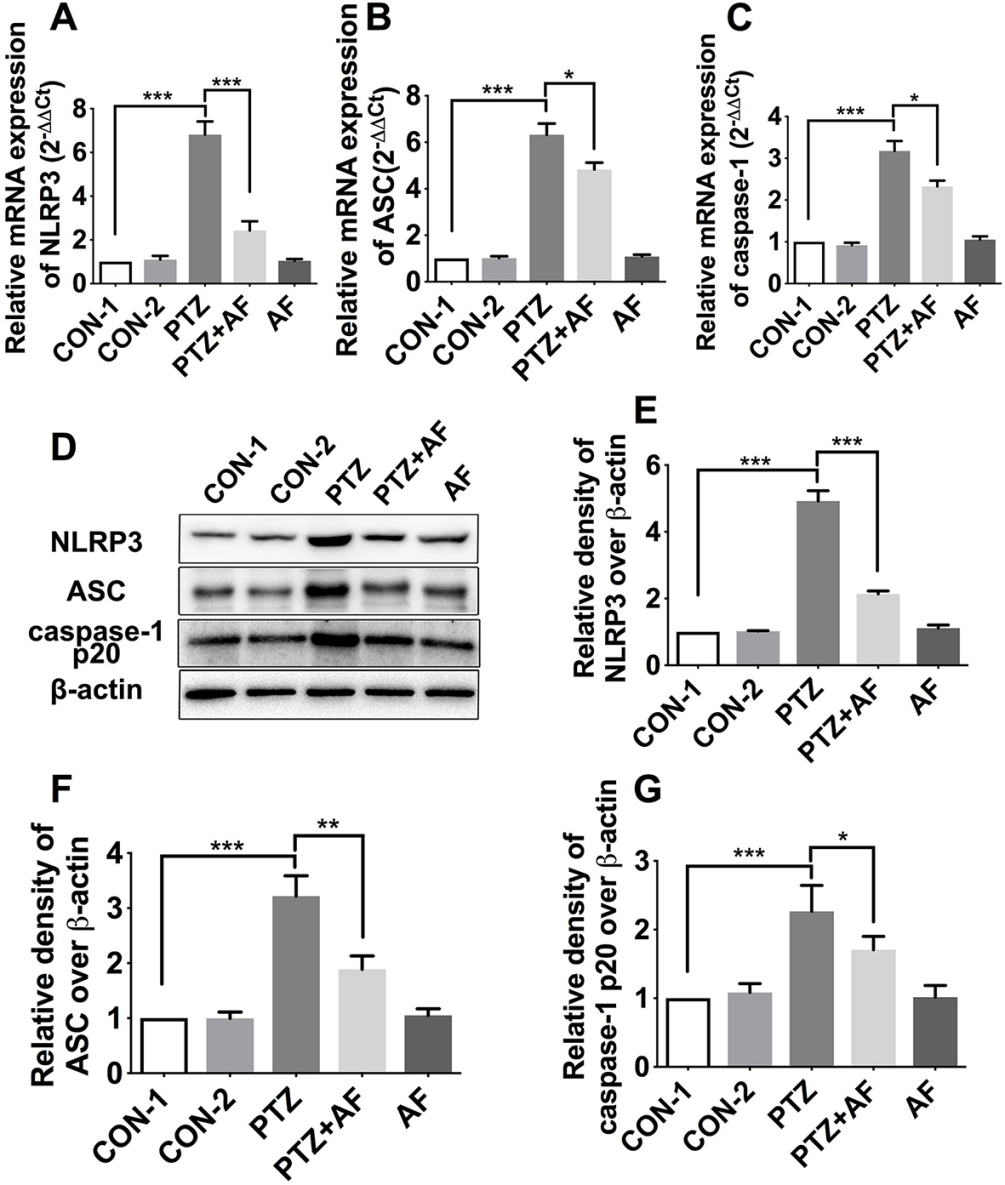
Effects of amentoflavone on the expression of hippocampal NLRP3 inflammasome in PTZ-induced kindling mice. **(A**–**C)** mRNA expression level of NLRP3 inflammasome (NLRP3, ASC, and caspase-1) in different groups (*n* = 6 mice per group, *Student’s t test*). **(D)** Representative immunoblots of NLRP3, ASC, and caspase-1 p20 proteins in different groups. **(E**–**G)** Semi-quantitative analysis of the relative levels of NLRP3, ASC, and caspase-1 p20 by densitometric analysis (*n* = 6 mice per group, *Student’s t test*). Error bars represent mean ± s.d. *, **, *** represent *p* < 0.05, *p* < 0.01, *p* < 0.001, respectively. PTZ, pentylenetetrazole; AF, amentoflavone.

As a potential target for the treatment of epileptogenesis, NLRP3 inflammasome has been demonstrated to be associated with neuroinflammation and epilepsy. The mRNA and protein levels of NLRP3 inflammasome complexes in the hippocampus were respectively detected by real-time PCR and Western blot analysis. Compared to the control group, NLRP3, ASC, and caspase-1 mRNA for mice in the PTZ group were significantly increased (*p* < 0.001); however, this increase due to PTZ-induced kindling was significantly prevented by amentoflavone treatment (*p* < 0.05). Amentoflavone alone did not influence the mRNA of NLRP3 inflammasome (*p* > 0.05; **Figures 4A–C**). Similar to mRNA levels, protein levels of NLRP3, ASC, and caspase-1 in the PTZ group were significantly higher than those in the control group (*p* < 0.001). Amentoflavone treatment could significantly inhibit NLRP3, ASC, and caspase-1 expression induced by PTZ-induced kindling (*p* < 0.05), but when administered alone, amentoflavone did not influence the expression of NLRP3, ASC, and caspase-1 (*p* > 0.05; **Figures 4D–G**). These data suggest that PTZ-induced kindling activates the NLRP3 inflammasome and that amentoflavone inhibits NLRP3 inflammasome activation.

### Amentoflavone Decreases the Levels of Inflammatory Cytokines in the Hippocampus of PTZ-Induced Kindling Mice

**Figure 5:**
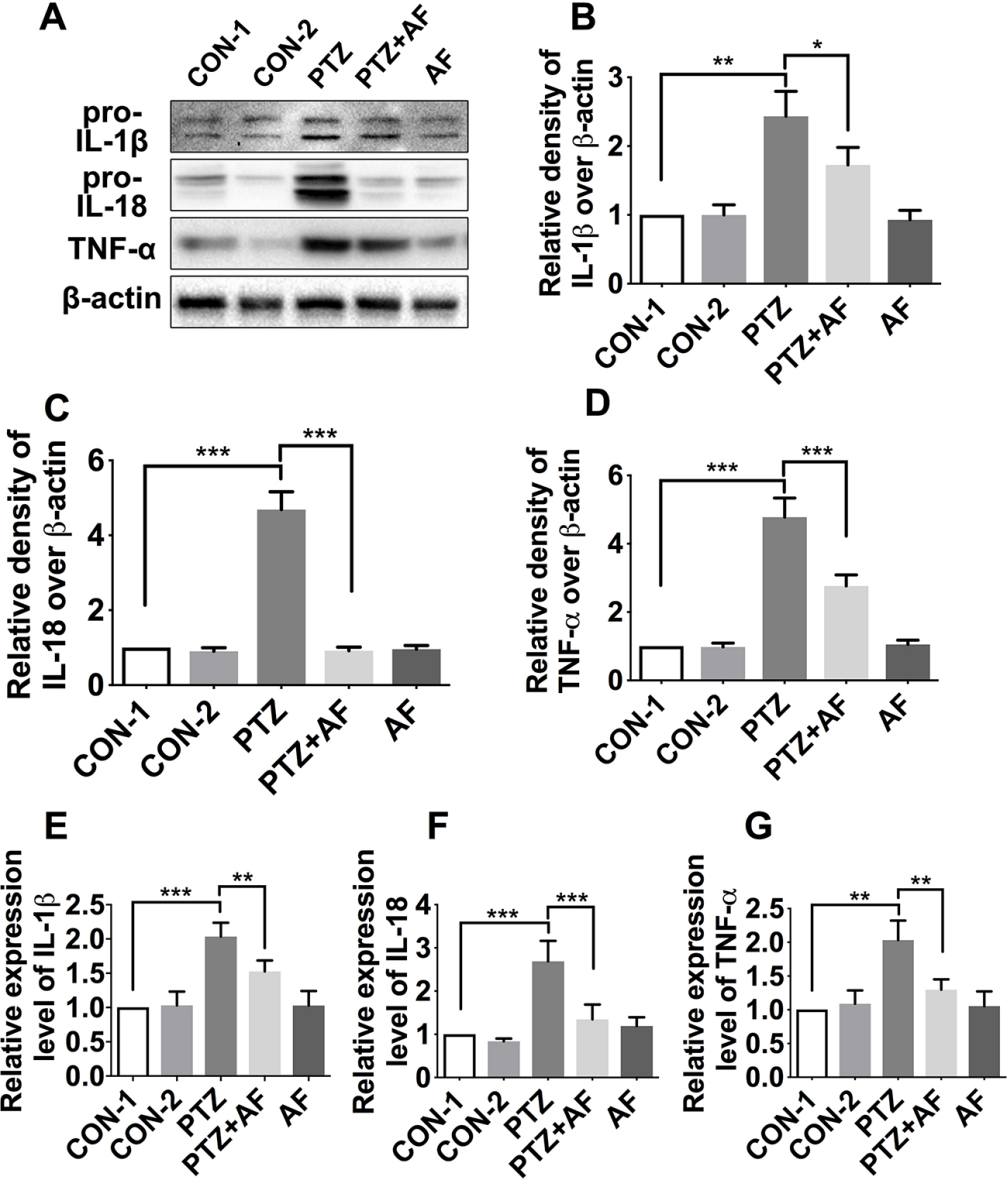
Effects of amentoflavone on the expression of inflammatory cytokines in the hippocampus by PTZ-induced kindling. **(A)** Representative immunoblots of IL-1β, IL-18, and TNF-α proteins in different groups. **(B**–**D)** Semi-quantitative analysis of the relative levels of IL-1β, IL-18, and TNF-α by densitometric analysis (*n* = 6 mice per group, *Student’s t test*). **(E**–**G)** Semi-quantitative analysis of the relative levels of IL-1β, IL-18, and TNF-α using ELISA (*n* = 9 mice per group, *Student’s t test*). Error bars represent mean ± s.d. *, **, *** represent *p* < 0.05, *p* < 0.01, *p* < 0.001, respectively. PTZ, pentylenetetrazole; AF, amentoflavone.

Inflammasome activation promotes the secretion of inflammatory cytokines such as IL-18, IL-1β, and TNF-α. To determine the effect of amentoflavone on pro-inflammatory cytokines in PTZ-induced kindling in mice, we detected the levels of IL-1β, IL-18, and TNF-α in the hippocampus *via* Western blot (**Figures 5A**). Compared to the control group, the levels of IL-1β, IL-18, and TNF-α were significantly increased in the PTZ group (*p* < 0.01). However, by treatment with amentoflavone, the expression of these inflammatory cytokines were significantly decreased (*p* < 0.05), but when administered alone, amentoflavone did not influence their expression. In fact, there was no statistical difference between the group treated with amentoflavone alone and the control group (*p* > 0.05; **Figures 5B–D**). Similar to the Western blot results, those of ELISA showed that the cytokines IL-1β, IL-18, and TNF-α were significantly higher in the PTZ group than in the control group (*p* < 0.001). However, amentoflavone treatment significantly inhibited IL-1β, IL-18, and TNF-α expression due to PTZ-induced kindling (*p* < 0.01), but when administered alone, amentoflavone did not influence the expression of these cytokines (*p* > 0.05; **Figures 5E–G**). These results indicate that amentoflavone inhibits NLRP3 inflammasome-mediated inflammatory processes in PTZ kindled mice.

### Amentoflavone Alleviated LPS-Induced Inflammation Response by Inhibiting NLRP3 Inflammasome in BV2 Microglia Cells

**Figure 6:**
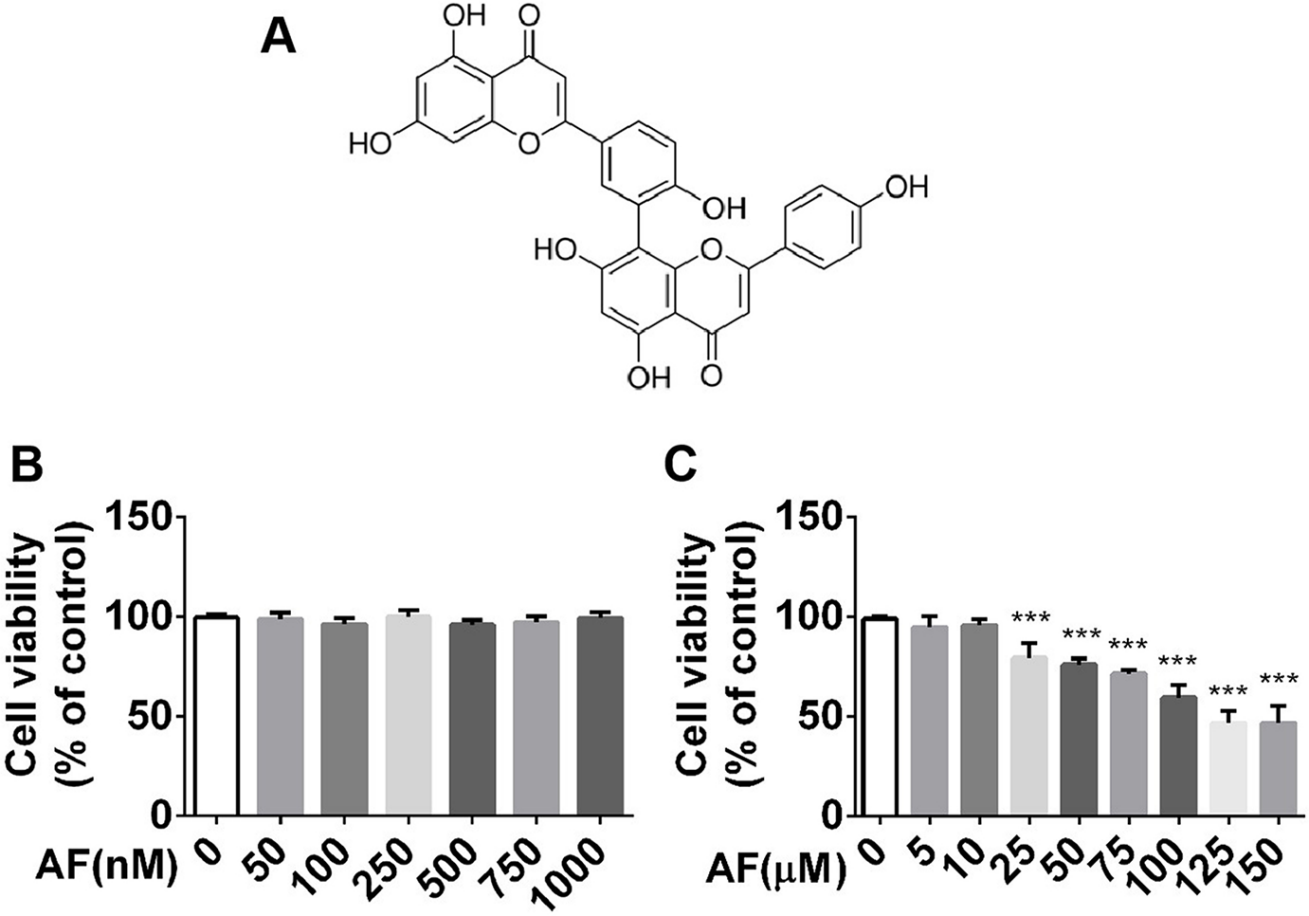
MTT analysis of the effect of amentoflavone on viability of BV2 microglial cells. **(A)** Structure of amentoflavone. **(B)** Viability of BV2 microglial cells treated with amentoflavone in graded concentrations (0, 50, 100, 250, 500, 750, or 1000 nM) was measured by MTT assay (one-way ANOVA followed by *Dunnett’s test*). **(C)** Viability of BV2 microglial cells treated with amentoflavone in graded concentrations (5, 10, 25, 50, 75, 100, 125, and 150 µM) was measured by MTT assay (one-way ANOVA followed by *Dunnett’s test*). Error bars represent mean ± s.d. Compared to control group, *** represents *p* < 0.001. All experiments were performed in triplicate. AF, amentoflavone.

Activated microglia can exacerbate brain inflammation, resulting in secondary damage that can cause or exacerbate seizures. Hence, we sought to evaluate the neuroprotective effects of amentoflavone using BV2 microglial cells. First, viability of BV2 microglial cells in graded concentrations of amentoflavone was determined by an MTT assay. Based on the results, amentoflavone did not exhibit toxicity at lower doses (*p* > 0.05; **Figures 6A, B**) and hardly impacted cell viability at higher doses when its concentration was lower than or equal to 10 µM (**Figure 6C**). Therefore, 10 µM amentoflavone was used in the subsequent study.

**Figure 7:**
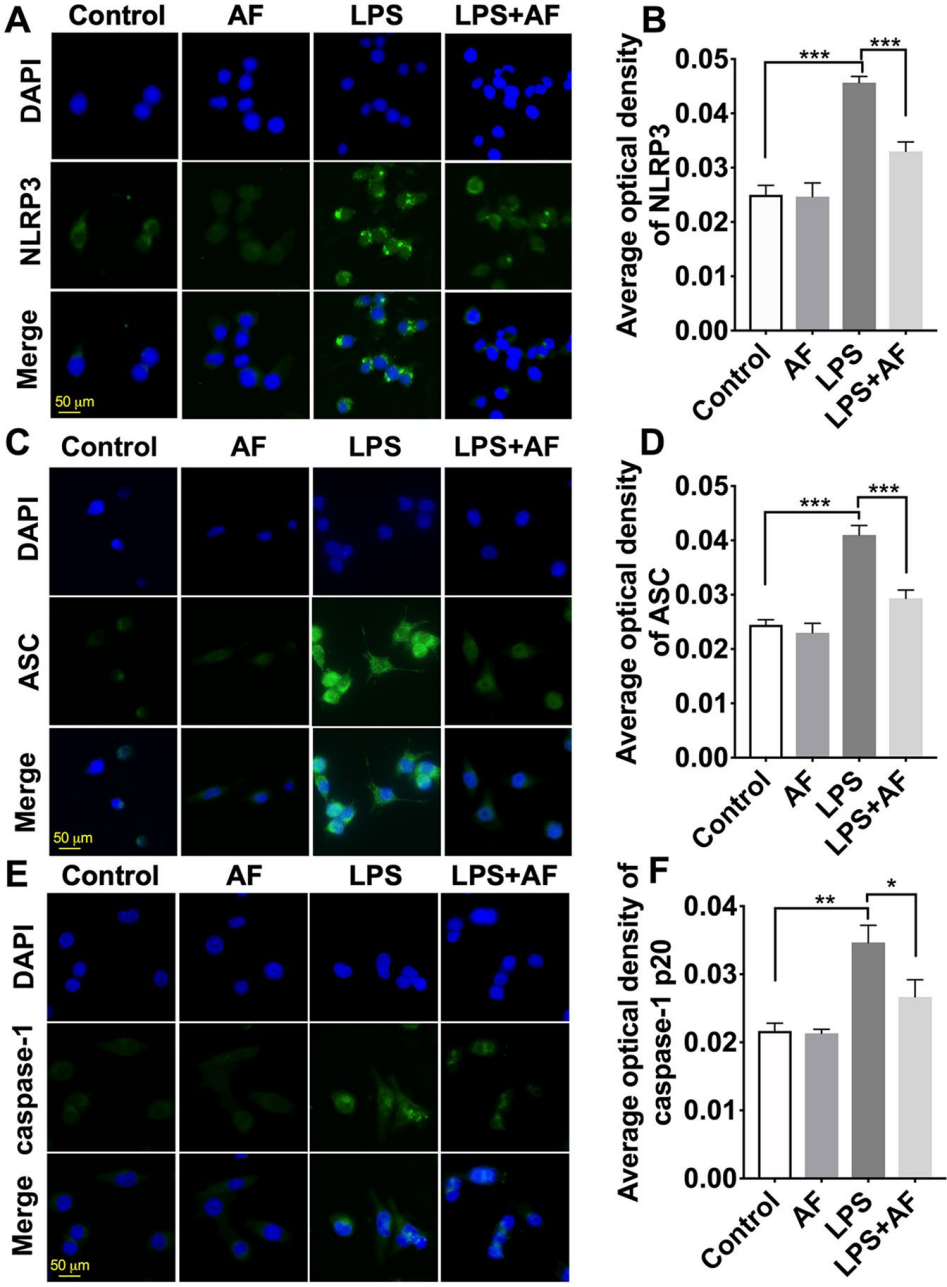
Effects of amentoflavone on the expression of NLRP3 inflammasome activated by LPS in BV2 microglial cells. **(A**, **C**, and **E )** Representative images of immunofluorescence staining of NLRP3, ASC, and caspase-1 in BV2 microglial cells. **(B**, **D**, and **F)** Semi-quantitative analysis of the relative levels of NLRP3, ASC, and caspase-1 by densitometric analysis (*Student’s t test*). Error bars represent mean ± s.d. *, **, *** represent *p* < 0.05, *p* < 0.01, *p* < 0.001, respectively. All experiments were performed in triplicate. LPS, lipopolysaccharide; AF, amentoflavone.

Immunostaining was performed with anti-NLRP3, anti-ASC, and anti-caspase-1 antibodies, and the morphology of cells was observed (**Figures 7A, C, E**). Compared to the control group, BV2 microglial cells stimulated with LPS displayed long, thin processes extending from their cell body. By pretreatment with amentoflavone and then treatment with LPS, this process was reduced and cell morphology appeared similar to those of control cells. In addition, NLRP3 inflammasome was activated by LPS in BV2 microglial cells. Fluorescence intensity of NLRP3, ASC, and caspase-1 was significantly enhanced by LPS (*p* < 0.01). Indeed, pretreatment with amentoflavone followed by LPS treatment significantly reduced the fluorescence intensity induced by LPS (*p* < 0.05), but when administered alone, amentoflavone did not influence the activation of NLRP3 inflammasome (*p* > 0.05; **Figures 7A–F**).

**Figure 8:**
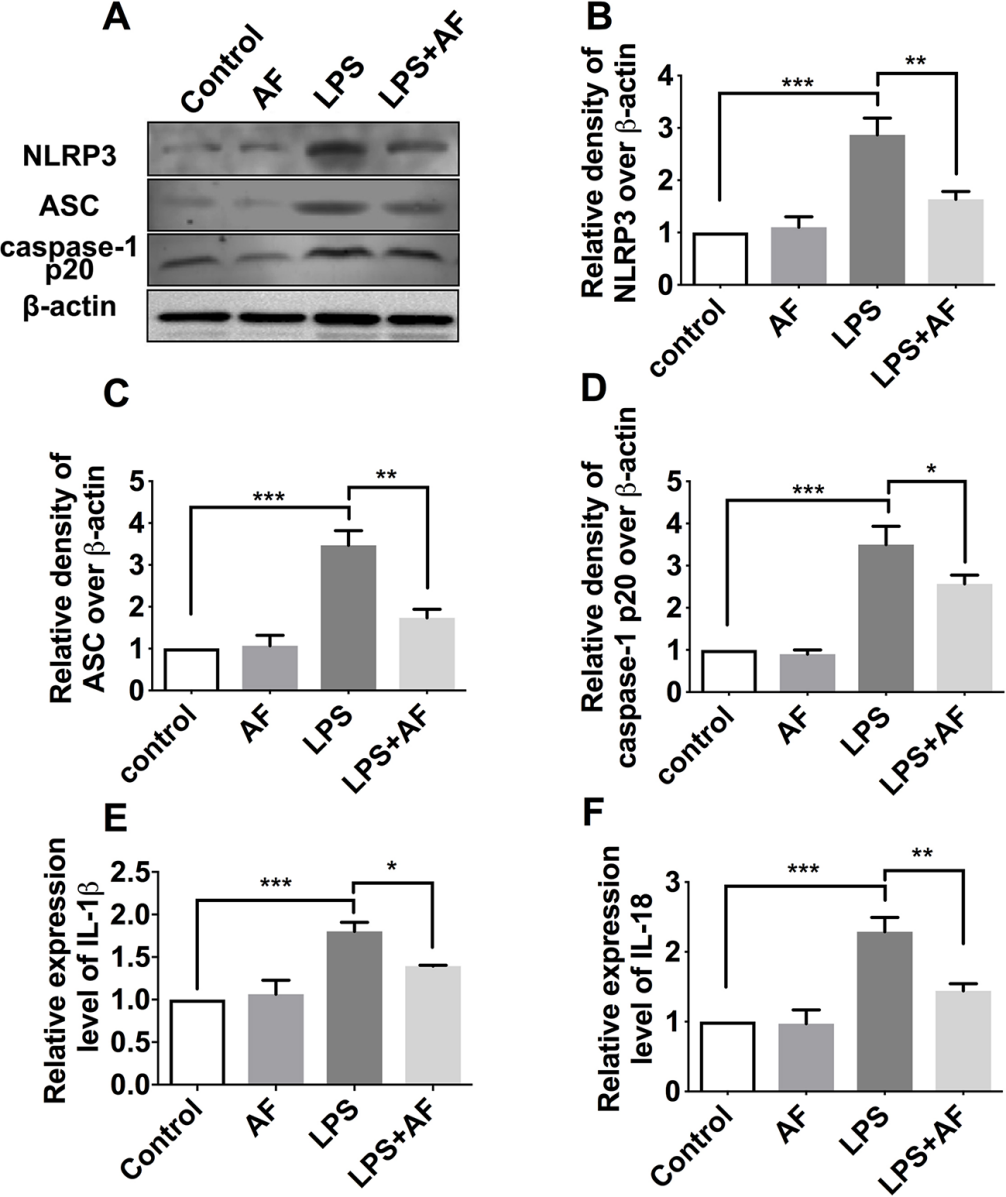
Effects of amentoflavone on the protein expression of NLRP3 inflammasome activated by LPS in BV2 microglial cells. **(A)** Representative immunoblots of NLRP3, ASC, and caspase-1 p20 proteins in different groups. **(B**–**D)** Semi-quantitative analysis of the relative levels of NLRP3, ASC, and caspase-1 p20 by densitometric analysis (*Student’s t test*). **(E** and **F)** Semi-quantitative analysis of the relative levels of IL-1β and IL-18 using ELISA (*Student’s t test*). Error bars represent mean ± s.d. *, **, *** represent *p* < 0.05, *p* < 0.01, *p* < 0.001, respectively. All experiments were performed in triplicate. LPS, lipopolysaccharide; AF, amentoflavone.

To further confirm these findings, the protein level of NLRP3 inflammasome complexes (NLRP3, ASC, and caspase-1) in BV2 microglial cells was detected by Western blot (**Figure 8A**). Stimulation with LPS was demonstrated to activate the NLRP3 inflammasome (*p* < 0.001); however, amentoflavone could significantly prevent the increase in NLRP3 inflammasome expression induced by this stimulation (*p* < 0.05; **Figures 8B–D**). In addition, the results of ELISA showed that the cytokines IL-1β and IL-18 were significantly higher in the LPS group than in the control group (*p* < 0.001). However, amentoflavone treatment significantly inhibited IL-1β and IL-18 expression due to LPS stimulation (*p* < 0.05), but when administered alone, amentoflavone did not influence the expression of these cytokines (*p* > 0.05; **Figures 8E, F**). These data suggest that amentoflavone modulates the LPS-induced activation of NLRP3 inflammasome in BV2 microglial cells *in vitro*.

## Discussion

In this study, we revealed that amentoflavone reduced seizure susceptibility, minimized cognitive dysfunction, and blocked the apoptosis of hippocampal neurons in PTZ-induced kindling mice. Amentoflavone could also decrease the levels of inflammatory cytokines induced by PTZ in the hippocampus, and this was found to be mainly dependent on the inhibition of NLRP3 inflammasome signaling activation. The neuroprotective effects that are dependent on this inhibition were also observed in BV2 microglial cells. Hence, these findings indicate that amentoflavone’s ability to affect epileptogenesis, relieve seizures, and exert neuroprotective effects might be attributable to the inhibition of neuroinflammation *via* NLRP3 inflammasome inactivation.

Epilepsy is a neurological disease mainly caused by the excessive excitation of neurons (Moshe et al., 2015; Hauser et al., 2018) and is usually accompanied by cognitive dysfunction, such as learning and memory disorders (Barr and Jones, 2016). The hippocampus, which is closely related to epilepsy, is a key structure for learning and memory in mammals (Bohbot and Corkin, 2007). Epilepsy can cause or aggravate damage (Samokhina and Samokhin, 2018) and apoptosis (Teocchi and D’Souza-Li, 2016) of hippocampal neurons. Here, we found that amentoflavone treatment delayed the PTZ-kindling acquisition process, rescued seizure susceptibility, and minimized behavior and cognitive deficits in PTZ-induced kindling mice with epilepsy; these results are similar to those seen for Parkinson’s disease (Cao et al., 2017). Amentoflavone treatment could significantly increase the ratio of Bcl-2/Bax and decrease the PTZ-induced kindling expression of cleaved caspase-3 in the hippocampus. Interestingly, amentoflavone can induce human breast cancer (Lee et al., 2013), ovarian cancer (Liu et al., 2017), and hepatocellular carcinoma apoptosis (Lee et al., 2018), and this may be due to the large doses used. Amentoflavone may also activate other signaling pathways, such as autophagy, p53 modulation (Park and Kim, 2019), and the inhibition of ERK1/2 (Lee et al., 2018) or JAK2/STAT3 (Wu et al., 2017) activation. In our study, we found that amentoflavone could reduce apoptosis induced by PTZ. This might be because it can relieve the inflammatory response and reduce the brain damage caused by epilepsy, thereby reducing apoptosis. These results suggest that amentoflavone relieves seizures, minimizes PTZ-induced cognitive dysfunction, and blocks hippocampal neuronal damage and apoptosis in mice with PTZ-induced kindling epilepsy.

Inflammation can promote neuronal excitability and epileptogenesis (Vezzani et al., 2015) and assist in reducing the seizure threshold in susceptible brain regions (Mazarati et al., 2017) (i.e., regions that may exacerbate seizures or increase their frequency). Neuroinflammation has been determined as both the cause and the result of epilepsy (Barker-Haliski et al., 2017), and is a promoter of epileptogenesis (Vezzani et al., 2016). Many studies have confirmed that inflammatory mediators and cytokines such as IL-1β can mediate neuronal cell loss (Varvel et al., 2016), contribute to related molecular and synaptic plasticity (Thome et al., 2018), and affect synaptic transmission and neuronal excitability (Vezzani and Viviani, 2015), thereby promoting epileptogenesis, and ultimately leading to seizures and recurrence (Vezzani et al., 2015). Amentoflavone affects the production of inflammatory mediators including inducible cyclooxygenase-2 (COX-2), nitric oxide synthase (NOS), TNF-α, and IL-6, which are mediated by NF-κB signal transduction pathways (Sakthivel and Guruvayoorappan, 2013; Yen et al., 2018). Here, we found that amentoflavone treatment decreased the levels of pro-inflammatory cytokines (IL-1β, IL-18, and TNF-α) due to PTZ-induced kindling in the hippocampus. As IL-1β and IL-18 are key inflammatory cytokines of inflammasome-dependent cytokines, amentoflavone may relieve seizures, minimize PTZ-induced cognitive dysfunction, and exert neuroprotective effects through the inflammasome pathways, which are consistent with the results of our previous research (Zhang et al., 2015).

Inflammasomes have been found to activate inflammatory processes, thereby causing a substantial release of inflammatory cytokines such as IL-1β, IL-18, and TNF-α, which can aggravate inflammation and lead to seizures (Lenart et al., 2016; Tzeng et al., 2018). As the most classic inflammasome, NLRP3 is critical for inflammation. Hence, negative regulation of NLRP3 inflammasome activity is essential for controlling epileptogenesis and reducing seizures (Meng et al., 2014; Jang et al., 2016; Lenart et al., 2016; Wang et al., 2017), and may therefore be a target for the treatment of epilepsy. Through our research, we found that amentoflavone treatment significantly inhibited the mRNA and protein levels of NLRP3, ASC, and caspase-1 caused by PTZ-induced kindling. In addition, the PTZ-induced activation of the NLRP3 inflammasome promoted the secretion of inflammatory cytokines such as IL-18, IL-1β, and TNF-α. However, these inflammatory cytokines were significantly decreased by amentoflavone treatment. These findings are similar to our previous results where amentoflavone was found to exert neuroprotective effects and effectively suppress NF-κB-induced inflammatory response (Zhang et al., 2015). These results indicate that NLRP3 inflammasome was activated in the hippocampus by PTZ-induced kindling and promoted the release of inflammatory cytokines. This effect was however inhibited by amentoflavone treatment.

Epilepsy is well known to result in microglial activation (Morin-Brureau et al., 2018), which can in turn aggravate brain inflammation, exacerbating seizures (Hiragi et al., 2018). The NLRP3 inflammasome is mainly expressed in the microglia and not in astrocytes or oligodendrocytes (Heneka et al., 2014). As the NLRP3 inflammasome can be activated by LPS in BV2 microglial cells (Chen et al., 2019), these cells can be used to study the neuroprotective effects of amentoflavone *in vitro*. Here, we found that amentoflavone significantly prevented the increase in NLRP3 inflammasome expression induced by LPS stimulation. This finding indicates that amentoflavone inhibits the LPS-induced activation of the NLRP3 inflammasome *in vitro*.

The mechanism whereby amentoflavone regulates NLRP3 inflammasome signaling is not fully understood. Although experimental animal and cell models have been used for elucidating these mechanisms, further experimental research and rigorous clinical investigation should be conducted *via* human studies.

In our study, PTZ-induced kindling increased seizure susceptibility, induced cognitive dysfunction, and increased apoptosis, the expression of NLRP3 inflammasome (NLRP3, ASC, and caspase-1), and inflammatory cytokines (IL-1β, IL-18, and TNF-α) *in vivo* and *in vitro*. By administering amentoflavone, however, these effects could be improved. These findings indicate that amentoflavone affects epileptogenesis and exerts neuroprotective effects by inhibiting the NLRP3 inflammasome, which mediates the inflammatory process in mice with PTZ-induced kindling and LPS-induced BV2 microglial cells. These results offer a new insight into the molecular mechanism of epilepsy and suggest that amentoflavone may be a promising therapeutic agent for the treatment of epilepsy.

## Data Availability

All datasets generated for this study are included in the manuscript.

## Ethics Statement

The protocol for using mice in this study was approved by the Animal Research Ethics Committee of Ningxia Medical University (2014-143).

## Author Contributions

TS and FW participated in the design of the study. SR and DW performed the experiments, analyzed the data, and wrote the manuscript. YF analyzed the data and wrote the manuscript. SL, KS, JH, PZ, XL, and XX performed the experiments. All authors read and approved the final manuscript.

## Funding

This study was supported by the National Natural Science Foundation of China (No. 81460208) and the Ningxia Natural Science Foundation of China (No. NZ13163).

## Conflict of Interest Statement

The authors declare that the research was conducted in the absence of any commercial or financial relationships that could be construed as a potential conflict of interest.

## Abbreviations

PTZ, pentylenetetrazole; NLRP3, nucleotide oligomerization domain (NOD)-like receptor protein 3; ASC, apoptosis-associated speck-like protein containing a caspase-activating recruitment domain; IL-1β, inflammatory interleukin-1β; IL-18, interleukin-18; TNF-α, tumor necrosis factor-α; MWM, Morris water maze; qRT-PCR, quantitative real-time PCR; ELISA, enzyme-linked immunosorbent assay; PBS, phosphate buffer saline; TBST, tris-buffered saline and Tween 20; RT, room temperature.
